# Phylogeography and population structure of the global, wide host-range hybrid pathogen *Phytophthora* × *cambivora*

**DOI:** 10.1186/s43008-023-00109-6

**Published:** 2023-02-23

**Authors:** Martin S. Mullett, Kris Van Poucke, Annelies Haegeman, Fran Focquet, Nicholas C. Cauldron, Brian J. Knaus, Marilia Horta Jung, Koji Kageyama, Ayaka Hieno, Hayato Masuja, Seiji Uematsu, Joan F. Webber, Clive M. Brasier, József Bakonyi, Kurt Heungens, Niklaus J. Grünwald, Thomas Jung

**Affiliations:** 1 Phytophthora Research Centre, Department of Forest Protection and Wildlife Management, Mendel University in Brno, Brno, Czech Republic; 2 Flanders Research Institute for Agriculture, Fisheries and Food (ILVO), Plant Sciences Unit, Merelbeke, Belgium; 3 Department of Botany and Plant Pathology, Oregon State University, Corvallis, OR USA; 4 Department of Horticulture, Oregon State University, Corvallis, OR USA; 5 River Basin Research Center, Gifu University, Gifu, Japan; 6 Forestry and Forest Products Research Institute (FFPRI), Tsukuba, Ibaraki Japan; 7 Department of Bioregulation and Biointeraction, Laboratory of Molecular and Cellular Biology, Tokyo University of Agriculture and Technology, Fuchu, Tokyo, Japan; 8 Forest Research, Alice Holt Lodge, Farnham, Surrey, UK; 9 Plant Protection Institute, Centre for Agricultural Research, ELKH, Budapest, Hungary; 10 Horticultural Crops Research Unit, United States Department of Agriculture, Agricultural Research Service, Corvallis, OR USA; 11 Phytophthora Research and Consultancy, Nussdorf, Germany

**Keywords:** Invasive pathogen, Hybridization, Polyploidy, Population genetics

## Abstract

Invasive, exotic plant pathogens pose a major threat to native and agricultural ecosystems. *Phytophthora* × *cambivora* is an invasive, destructive pathogen of forest and fruit trees causing severe damage worldwide to chestnuts (*Castanea*), apricots, peaches, plums, almonds and cherries (*Prunus*), apples (*Malus*), oaks (*Quercus*), and beech (*Fagus*). It was one of the first damaging invasive *Phytophthora* species to be introduced to Europe and North America, although its origin is unknown. We determined its population genetic history in Europe, North and South America, Australia and East Asia (mainly Japan) using genotyping-by-sequencing. Populations in Europe and Australia appear clonal, those in North America are highly clonal yet show some degree of sexual reproduction, and those in East Asia are partially sexual. Two clonal lineages, each of opposite mating type, and a hybrid lineage derived from these two lineages, dominated the populations in Europe and were predominantly found on fagaceous forest hosts (*Castanea*, *Quercus*, *Fagus*). Isolates from fruit trees (*Prunus* and *Malus*) belonged to a separate lineage found in Australia, North America, Europe and East Asia, indicating the disease on fruit trees could be caused by a distinct lineage of *P.* × *cambivora*, which may potentially be a separate sister species and has likely been moved with live plants*.* The highest genetic diversity was found in Japan, suggesting that East Asia is the centre of origin of the pathogen. Further surveys in unsampled, temperate regions of East Asia are needed to more precisely identify the location and range of the centre of diversity.

**Supplementary Information:**

The online version contains supplementary material available at 10.1186/s43008-023-00109-6.

## Introduction

Exotic plant pathogens have repeatedly invaded forests and agricultural ecosystems worldwide. Increased human activity, including both increases in travel and plant trade, have been implicated in their accelerated global spread (Brasier 2008; Fisher et al. 2012; Santini et al. 2013; Wingfield et al. 2015). Prominent examples resulting in the deaths of tens of millions of trees include the spread of chestnut blight in the US; Dutch elm disease across Central Asia, Europe and North America; *Phytophthora cinnamomi* worldwide; the sudden oak death and sudden larch death pathogen in the US and Europe; and ash dieback in Europe (Brasier and Webber 2010; Grünwald et al. 2012; Landolt et al. 2016; Rigling and Prospero 2018; Shakya et al. 2021; Brasier et al. 2021). These invasions often eliminate foundation species substantially changing the plant community structure and function of ecosystems, which in turn can obliterate a forest’s ability to mitigate climate change (Seidl et al. 2018). Thus, understanding the evolutionary history, sources of potential migrants, and geographic origin of invasive pathogens will inform forest management and control strategies.

*Phytophthora* × *cambivora* (Petri) Buisman, originally named *Blepharospora cambivora* by Petri (1917), later transferred into *Phytophthora* by Buisman (1927), and classified as a hybrid by Jung et al. (2017a, b) is an invasive pathogen of broad concern. It is the principal causal agent of ink disease of sweet chestnut (*Castanea sativa* Mill.), together with *P. cinnamomi* Rands. The pathogen primarily infects the root system causing bark necroses which can spread to the collar and lower trunk resulting in extensive cortical lesions with black phloem exudates which also often stain the surrounding soil, giving rise to the common name of the disease (Vettraino et al. 2005; Jung et al. 2018b). Above-ground symptoms include wilting, chlorosis and microphylly (Vettraino et al. 2005; Jung et al. 2018b). Whilst most damaging and well known from sweet chestnut, *P.* × *cambivora* causes root and collar rots, aerial stem cankers, crown rots and severe mortality of a wide range of hosts, particularly members of the *Fagaceae* and many fruit trees in the *Rosaceae* and other horticultural species (Erwin and Ribeiro 1996; Jung et al. 1996, 2000, 2013, 2016, 2018b; Jung 2009). It has been found on over 40 host species across Europe, North America, Australia, parts of South America, Asia, as well as in numerous African countries (Erwin and Ribeiro 1996; CABI 2017). Severe damage to sweet chestnut was caused by *P.* × *cambivora* in the nineteenth and early twentieth centuries and since the 1990s a dramatic resurgence of ink disease has occurred, mainly in southern Europe, in some cases limiting the establishment of new groves of sweet chestnut (Vannini and Vettraino 2001; Vettraino et al. 2001, 2005; Fleisch 2002; Robin et al. 2006; Jung et al. 2018b). The involvement of *P.* × *cambivora*, particularly since *c*. 2000, in the widespread declines of beech (*Fagus sylvatica*) and oak (*Quercus* spp.) stands in central and northern Europe, the unexpected detection of the pathogen causing aerial cankers and xylem and shoot infections on beech (Brown and Brasier 2007; Černý et al. 2006; Corcobado et al. 2020; Jankowiak et al. 2013; Jung 2009; Jung et al. 2000, 2005, 2006, 2018a, b; Nechwatal et al. 2011; Telfer et al. 2015), reports on chinquapin (*Chrysolepis chrysophylla*) in North America (Saavedra et al. 2007), and persistent root and crown rot problems on fruit trees (*Prunus* spp. and *Malus* spp.) (Wilcox and Mircetich 1985; Erwin and Ribeiro 1996), illustrate the longstanding and serious economic and ecological impacts of the taxon.

*Phytophthora* × *cambivora* was probably among the first damaging invasive *Phytophthora* species to be introduced to Europe and North America, assumed to have arrived in Europe in the eighteenth century, yet almost nothing is known about its origin and mode of arrival (Crandall 1950; Peace 1962). In a rare population study of the species Oudemans and Coffey (1991) found all isolates from Europe to have a single multilocus isozyme genotype, whilst those from Australia were more variable, possibly suggesting an Australasian origin of the pathogen. Importations of plant pathogens are often limited in number of individuals and genetic variability when compared to populations in their centre of origin as a result of genetic bottlenecks (Goodwin 1997) and the rapid emergence of asexual clones of higher fitness in the new environment (Brasier 1995). For heterothallic species only one mating type may be introduced or survive, prohibiting sexual recombination and resulting in asexually reproducing clonal lineages (Goodwin 1997). Alternatively, certain clones may dominate due to particularly high fitness, even in the context of frequent sexual reproduction after introduction, giving the impression of a stronger introductory genetic bottleneck than may have truly occurred (Brasier and Kirk 2000). In contrast, native populations in their centre of origin often contain both mating types, reproduce sexually, and have high levels of genetic diversity. Some of the world’s most damaging *Phytophthora* pathogens such as *P. infestans, P. cinnamomi*, and *P. ramorum* exhibit this pattern (Goss et al. 2014; Jung et al. 2021; Shakya et al. 2021). For example, the potato late blight pathogen *P. infestans*, cause of the Irish potato famine, occurs as a diverse sexually recombining population in one of its hypothesized centres of origin in Mexico while clonal lineages cause devastating disease epidemics in Europe and North America (Cooke et al. 2012; Goss et al. 2014). However, other species do not strictly comply with this pattern, having populations with both mating types and high levels of genetic diversity, presumably from sexual reproduction, even in regions where they have been introduced, for example *P. capsicii* in the USA and South Africa (Lamour et al. 2012). Although the existence of the two mating types in *Phytophthora* has been known for 100 years, their exact functioning and molecular basis was unclear (Ashby 1922; Haasis and Nelson 1963). Sexual reproduction in the genus is under hormonal control and each mating type responds to the hormones, acyclic oxygenated diterpenes termed α1 and α2, produced by the opposite mating type to produce oospores (Tomura et al. 2017). Nonetheless, in several heterothallic *Phytophthora* species pure single isolate cultures have been found to self and produce oospores in response to a range of stimuli such as fungicides, long-term culture, compounds produced by root exudates, bacteria, and fungi (Mukerjee and Roy 1962; Brasier 1971, 1972; Ko 1981; Groves and Ristaino 2000; Jayasekera et al. 2007), including A2s of *P.* × *cambivora* (Brasier 1975). In addition, a change in mating type has been recorded in some heterothallic species, usually from A2 to A1 (Ko 1981; Ann and Ko 1989; Chandelier et al. 2014), and several self-fertile *P.* × *cambivora* isolates have changed to A2 after longterm storage (T. Jung, unpublished results). Recently, the first oomycete mating type locus was identified, with one mating type homozygous and the other heterozygous (Dussert et al. 2020). This is consistent with the Sansome (1980) model that one *Phytophthora* mating type (A2) is heterozygous and the other (A1) is homozygous; and that somatic segregation of the homozygote from the heterozygote type is restricted by chromosomal reciprocal translocation. Sansome (1980) also showed that the translocation was present in *P.* × *cambivora*. These findings help further explain the potential to change from the A2 to the A1 mating type, and indicate that even if a single mating type of an exotic heterothallic *Phytophthora* species is introduced to a region, sexual reproduction may still occur, either via stimuli that promote selfing or transformation to the other mating type. All considered, limited information is available on the behaviour, population structure and origin of the heterothallic *P.* × *cambivora* across its distribution range.

Interspecific hybridization is well known as an important evolutionary driving force in plants, animals and, increasingly, in fungal pathogens (Brasier 2001) and the genus *Phytophthora,* where six of the 12 clades are known to include hybrid taxa (Chen et al. 2022; Soltis et al. 2010; Soltis and Soltis 2000; Van Poucke et al. 2021). Hybridization can be homoploid, where the ploidy of the hybrid remains the same as that of the parents, or polyploid, where the entire genomes of each parent are retained and genome doubling occurs in the hybrid (Soltis and Soltis 2009). When polyploid hybridization is between different species it is known as alloploidy, whereas when it occurs between populations of the same species it is known as autopolyploidy (Soltis and Soltis 2000, 2009). Each of these hybridization processes have different genetic consequences for the resulting hybrids (Soltis and Soltis 2000, 2009). Hybridization is thus often accompanied by polyploidization, and although still poorly understood, these processes can infer a fitness advantage and increase adaptability, essential traits influencing the invasiveness of a species (Ellstrand and Schierenbeck 2000; Schierenbeck and Ellstrand 2008; Soltis et al. 2010). Polyploid hybrids can be better suited to specific environments and can exhibit an extended host range and enhanced vigour compared to their parents (Brasier et al. 1999; Bertier et al. 2013; Burgess 2015; Jung et al. 2017a, b). Alder decline, caused by *P.* × *alni,* is a recent example of a polyploid hybrid *Phytophthora* wreaking widescale ecological destruction (Husson et al. 2015). Recently Jung et al. (2017b) classified *P.* × *cambivora* as an interspecific hybrid due to multiple heterozygous positions in ITS, β-tubulin, and *HSP90* gene sequences as well as evidence from cloned β-tubulin, and *HSP90* sequences. Van Poucke et al. (2021) also considered the species to be an alloploid hybrid based on its large genome size determined by flow cytometry, comparison of the genome size and the number of GBS loci found, and the presence of a large number of triallelic loci. However, its ploidy level and origins remain unclear.

Overall information on the origins, behaviour, population structure and ploidy levels of *P.* × *cambivora* worldwide remains limited. Although potentially native to East Asia, isolates of *P.* × *cambivora* from the region have been scarce, a situation improved by our 2017 survey of *Phytophthora* diversity in natural ecosystems of Japan during which numerous isolates were obtained with both morphological and ITS sequence resemblance to *P.* × *cambivora.* Based on this survey, we studied the global population structure of the pathogen including isolates from Europe, North and South America, Australia, and East Asia. We used genotyping-by-sequencing (GBS) to obtain genome-wide single nucleotide polymorphisms (SNPs) to characterize the global population structure of *P.* × *cambivora* and its reproductive mode across continents and infer a potential centre of origin. Recent and ancient hybridization events, variation in ploidy and the traces these events have left in the genome are discussed. This work provides novel insights into the emergence of pathogens through hybridization and migration.

## Materials and methods

### Isolate selection and DNA extraction

*Phytophthora* × *cambivora* sensu lato isolates were selected from across the pathogen’s reported range, covering North and South America, Australia, Asia, and Europe (Additional file 1: Table S1). Isolate selection was particularly focused on Europe, where the pathogen is widespread and problematic, and Japan, where a 2017 *Phytophthora* survey revealed a large number of isolates with ITS sequence similarities above 99% and morphological resemblance to *P.* × *cambivora*. Although sampling from certain continents was limited (e.g. only USA in North America, Chile in South America, predominantly Japan in East Asia) the isolates were taken to be representative of the region. Nine *P.* × *alni* isolates were included as an outgroup.

Mycelium for DNA extraction was obtained by growing isolates in 17 ml 5% clarified V8 juice broth for one week at 20 °C in a shake culture. Mycelium was then rinsed thoroughly with sterile distilled water and vacuum-dried on a Whatman No 1 filter (Maidstone, UK). DNA was extracted using the Nucleospin Plant II kit (Macherey–Nagel, Düren, Germany) with extraction buffer PL1, according to the manufacturer’s protocol, and eluted into 50 μl.

### Genotyping-by-sequencing, read processing, SNP calling, and data filtering

GBS libraries were prepared following the approach of Elshire et al. (2011) and Poland et al. (2012), specifically using the detailed method described in Van Poucke et al. (2021). Briefly, this consisted of digestion of genomic DNA with *Pst*I and *Hpa*II, annealing of adaptors and barcodes, and fragment amplification. Sixty-four to 80 isolates, each with a unique barcode, were pooled and paired-end sequenced (2 × 150 bp) using an Illumina HiSeq4000 (San Diego, CA, USA).

The sequences were pre-processed using the custom made pipeline of Van Poucke et al. (2021), available at https://gitlab.com/ahaegeman/GBS_Phytophthora and at Zenodo with https://doi.org/10.5281/zenodo.3363287. This pipeline consisted of (1) demultiplexing of reads using GBSX v1.1.5 (Herten et al. 2015), (2) trimming of adapters using cutadapt v1.16 (Martin 2011) and FastX toolkit v0.0.14, (3) merging of forward and reverse reads with PEAR v0.9.8 (Zhang et al. 2014), and (4) quality filtering using FastX toolkit, prinseq-lite (Schmieder and Edwards 2011), OBITOOLS v1.2.5 (Boyer et al. 2016) and pairfq 0.14. A custom database of prokaryotes, fungi, the human genome (build 38), and all available *Phytophthora* genomes was created (Van Poucke et al. 2021) and used in a local BLAST search of the GBS loci. Isolates with more than 450 GBS tags with significant BLASTn hits (E < 1e−4) to non-*Phytophthora* sequences were considered potentially contaminated and removed from the dataset.

Subsequently a reference-based locus identification approach used BWA-MEM 0.7.15 (Li 2013) to map the pre-processed GBS reads to the *P.* × *cambivora* genome (isolate TJ0032, GCA_000443045.1) (Feau et al. 2016). The resulting sam file was converted to bam format, sorted, and indexed using samtools 1.9 (Li et al. 2009). As the *P.* × *cambivora* genome is large and consists of over 70,000 contigs it was divided into 20 blocks of contigs using seqtk-1.0 (https://github.com/lh3/seqtk). The GBS reads matching the contigs in each of the 20 genome blocks were extracted from the mapped bam file using GATK Reorder and variants called using GATK HaplotypeCaller v4.0.12.0 on each of the blocks (McKenna et al. 2010). The 20 individual gvcf files for each isolate were then combined into a single file using GATK CombineGVCFs. VCFR 1.10.0 (Knaus and Grünwald 2017) was used to remove loci with a read depth of < 5 and > 70 and loci with > 80% missing data, after which all individual isolate vcf files were combined using vcftools-0.1.15 (Danecek et al. 2011). Indels and non-polymorphic sites were removed and only bi-allelic SNPs retained using VCFR.

### Analysis of genetic structure

Alleles in linkage disequilibrium can adversely affect many population clustering approaches and at best are redundant (Abdellaoui et al. 2013; Malomane et al. 2018; Calus and Vandenplas 2018; Privé et al. 2020). Therefore, for population structure analyses, linkage disequilibrium (LD) based SNP pruning and minor allele frequency (MAF) filtering were conducted in plink 1.9 (Chang et al. 2015; www.cog-genomics.org/plink/1.9/) using a 50 SNP window size, a 5 SNP step size, and a variance inflation factor [(1/(1 − r^2^)] of 1.5 (setting –indep 50 5 1.5) and a MAF of 5%. Additionally, only SNPs with < 5% missing data were retained. Four complementary population analysis methods were implemented: (1) STRUCTURE, (2) principal components analysis (PCA), (3) discriminant analysis of principal components (DAPC), and (4) maximum likelihood (ML) trees.

STRUCTURE 2.3.4 (Falush et al. 2003) implements a Bayesian, model-based clustering algorithm to assign individuals to a specified number of clusters (K), maximizing Hardy–Weinberg equilibrium and minimizing linkage disequilibrium within the clusters (Pritchard et al. 2000). To estimate the optimal number of clusters, 10 independent runs of K = 1–15 were carried out in STRUCTURE using no priors (i.e. no information on geographical location or host was provided). The Python utility StrAuto was used to parallelize the analysis (Chhatre and Emerson 2017). Each run had a burn-in of 100,000 iterations followed by 500,000 data-collecting iterations, used a model of correlated allele frequencies and with admixture among populations allowed. The optimal value of K was assessed using the ΔK method of (Evanno et al. 2005) in CLUMPAK (Kopelman et al. 2015), which was also used to align all optimum K STRUCTURE runs to the permutation with the highest H-value. The DISTRUCT version 1.1 program (Rosenberg 2004) was used to visualize the CLUMPP output.

To complement the Bayesian approach implemented in STRUCTURE, PCA, a method that makes no genetic assumptions (e.g. population model or data structure), was conducted in the R package adegenet 2.1.3 (Jombart and Ahmed 2011). To extend the PCA, a DAPC was also conducted in adegenet 2.1.3 (Jombart et al. 2010; Jombart and Ahmed 2011). The method is particularly suited to identifying clusters (K) of genetically related individuals as it minimizes variation within groups and maximizes variation between groups (Jombart et al. 2010). A sequential K-means procedure followed by an assessment of the Bayesian information criterion (BIC) to assess the optimal number of clusters precedes the DAPC analysis itself. Cross-validation was used to determine the optimal number of principal components retained in the analysis (Jombart and Collins 2015).

Phylogenetic trees are known to be inadequate at placing reticulate taxa, i.e. those derived from hybridization, introgression, or lateral gene transfer between two independent lineages (Dowling and Secor 1997; Gauthier and Lapointe 2007). Nevertheless, in some cases reticulate phylogenies can be partially revealed by traditional phylogenetic inference methods which can offer insights into the clustering of hybrid individuals if interpreted with caution (Posada and Crandall 2002). To this end RAxML v8.2.12 (Stamatakis 2014) was used to produce a maximum likelihood (ML) phylogenetic tree with the full dataset (i.e. prior to LD pruning and MAF filtering), with *P.* × *alni* used as an outgroup. All invariant SNPs were removed from the dataset using ascbias (https://github.com/btmartin721/raxml_ascbias). The GTRCAT model without rate heterogeneity with a correction for ascertainment bias (ASC_GTRCAT), together with the Lewis correction for ascertainment bias (asc-corr = lewis) were used and 1000 bootstrap replicates were performed. Figtree 1.4.4 was used to visualize the output (Rambaut 2018). For comparison a dendrogram was constructed using the Unweighted Pair Group Method with Arithmetic Mean (UPGMA), bitwise distance, and 100 bootstraps using poppr 2.9.3 (Kamvar et al. 2014) and ape 5.4-1 (Paradis and Schliep 2019).

### Mating type and inferring the mode of reproduction

Isolates were paired with known tester strains of *P.* × *cambivora* TJ0029 (A2 mating type) and TJ0030 (A1) to determine their mating type. Plugs (5 mm diam.) were cut from actively growing V8-juice agar (V8A) cultures and placed on opposite sides of 45 mm Petri dishes containing clarified V8A and incubated at 20 °C in the dark. Oogonia formation was assessed after four weeks under a light microscope at ×80 magnification (Jung et al. 2011, 2017b).

The predominant mode of reproduction was inferred using the Index of Association (I_A_), a measure of linkage disequilibrium (Brown et al. 1980; Milgroom 1996). The I_A_ was first calculated on 1000 simulated datasets with 0, 50, or 100% linkage representing sexual, semiclonal, and clonal populations. The simulated dataset contained 6767 loci (analogous to the *P.* × *cambivora*-related dataset) and was constructed using adegenet 2.1.3 (Jombart and Ahmed 2011); the I_A_ was calculated in poppr 2.9.3 (Kamvar et al. 2014) on one third of the loci (i.e. 2256 loci). As a single SNP is unlikely to produce a new multilocus genotype, particularly as genotyping error and missing data are common in high throughput sequencing data, individual genotypes were collapsed into multilocus lineages using the average neighbour algorithm (genetic distance cutoff of 0.02900025) (Kamvar et al. 2015) implemented in poppr. The I_A_ was calculated on the mulitilocus lineage dataset for each regional population (Australia, East Asia, Europe, and North America) and compared to that of the simulated datasets (Tabima et al. 2018). The South American population was excluded due to its small sample size. After testing the data for normality using the Shapiro–Wilk’s test a Kruskal–Wallis rank sum test and a posthoc rank comparison was conducted in R (R Development Core Team 2020).

### Hybridization analysis

Phylogenetic networks are more appropriate than phylogenetic trees for revealing relationships between reticulate taxa when recombination is suspected (Posada and Crandall 2001). SplitsTree v4.16.2 (Huson and Bryant 2006) was used to construct a phylogenetic network using the LD pruned and MAF filtered *P.* × *cambivora*-related only dataset implementing the neighbour-net and equal angle algorithms using uncorrected *p*-distances with heterozygous ambiguities averaged and normalized.

Nodes in implicit networks, such as those generated by Splitstree, do not represent ancestral taxa, whereas those in explicit networks do (Solís-Lemus and Ané 2016). For explicit network generation under the multispecies network coalescent (MSNC) Phylonetworks (Solís-Lemus and Ané 2016; Solís-Lemus et al. 2017) was used. Two representative isolates were chosen from each group (Additional file 1: Table S1), together with *P.* × *alni* as an outgroup, and concordance factors (CF) generated from the LD pruned and MAF filtered SNP dataset using the novel approach of Olave and Meyer (2020). A species tree was reconstructed under the multispecies coalescent (MSC) using the SVDquartets program (Chifman and Kubatko 2014) implemented in PAUP* version 4a168 (Swofford 2021). This species tree was used as the starting point for SNaQ (Solís-Lemus and Ané 2016), implemented in Phylonetworks, which was used to estimate the best network with a range of possible hybrid nodes allowed (from 0 to 6). Ten independent SNaQ searches were performed for each number of hybrid nodes tested, retaining those with the highest pseudolikelihood value.

To complement the estimates of ancestry coefficients provided by the population clustering methods and the results of the phylogenetic networks, a formal test of hybridization based on site pattern frequencies was implemented in HyDe (Blischak et al. 2018). HyDe considers a rooted, four-taxon network including an outgroup, in this case *P.* × *alni*, and a triplet of ingroup populations to detect hybridization based on phylogenetic invariants arising under the coalescent model (Blischak et al. 2018). An advantage over Patterson’s D-statistic (Patterson et al. 2012), popularly known as the ABBA-BABA test, is that it intrinsically accommodates multiple individuals per population while at the same time estimating the inheritance parameter, γ, that quantifies the genomic contributions of the parents to the hybrid (Kong and Kubatko 2020). All possible triplet combinations (i.e. using all 12 population groups) were tested and hypotheses considered significant at α < 0.05 after a Bonferonni correction with γ between 0 and 1 and Z-scores > 3.

### Ploidy investigation

Ploidy was inferred from GBS data using a number of methods. Gbs2ploidy 1.0 (Gompert and Mock 2017) was used to infer ploidy based on allelic ratios of heterozygous SNPs and to group isolates by ploidy level. The ratios of allele depths at heterozygous positions were also plotted to infer ploidy using vcfR 1.12.0 (Knaus and Grünwald 2017, 2018). The full dataset (i.e. prior to LD pruning and MAF filtering) was used with indels removed but with non-bi-allelic alleles retained. Diploids are expected to have alleles in a ratio of 1:2, triploids in a ratio of 1:3 (or 2:3) and tetraploids in a ratio of 1:4. The plots were organized by population group. Chromosome specific ploidy levels were not investigated due to the unassembled nature of the *P.* × *cambivora* reference genome and very high number of scaffolds (Feau et al. 2016).

## Results

### Genotyping-by-sequencing

A total of 296 *P.* × *cambivora*-related isolates from 26 countries were included in the study. An additional nine isolates of *P.* × *alni* were used as an outgroup. After removing loci with > 80% missing data and indels, and retaining only biallelic polymorphic SNPs, 408,666 SNPs remained in the *P.* × *cambivora*-related and *P.* × *alni* dataset, with 381,021 in the *P.* × *cambivora*-related dataset. After LD pruning, MAF filtering and removing loci with over 5% missing data 6,767 SNPs were retained in the final *P.* × *cambivora*-related dataset.

### Populations are strongly structured by continent

**Fig. 1:**
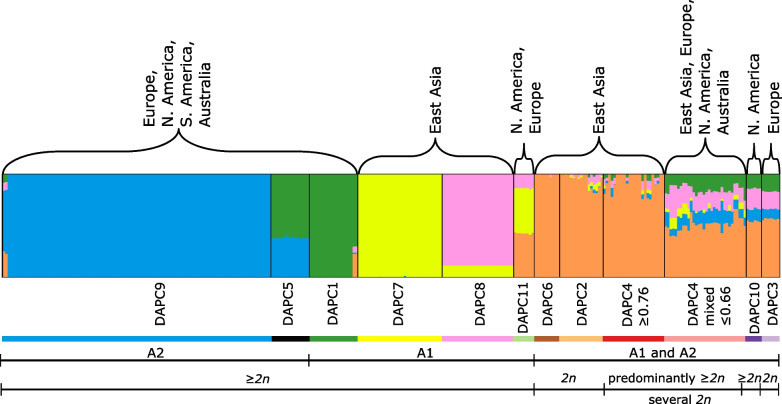
Bayesian clustering of *P.* × *cambivora*-related isolates using STRUCTURE at K = 5. Each isolate is represented by a vertical line partitioned into coloured sections that represent the isolate’s estimated membership fractions in each cluster. Black lines separate isolates from different DAPC groups (see main text for details). The horizontal colour bar below the DAPC group name represents the colour used for that group in maps and supplementary figures. The mating type (A1 or A2) and ploidy level (diploid = *2n* or higher) of each group is given below the plot and the geographic distribution of the group is given above the plot

**Fig. 2:**
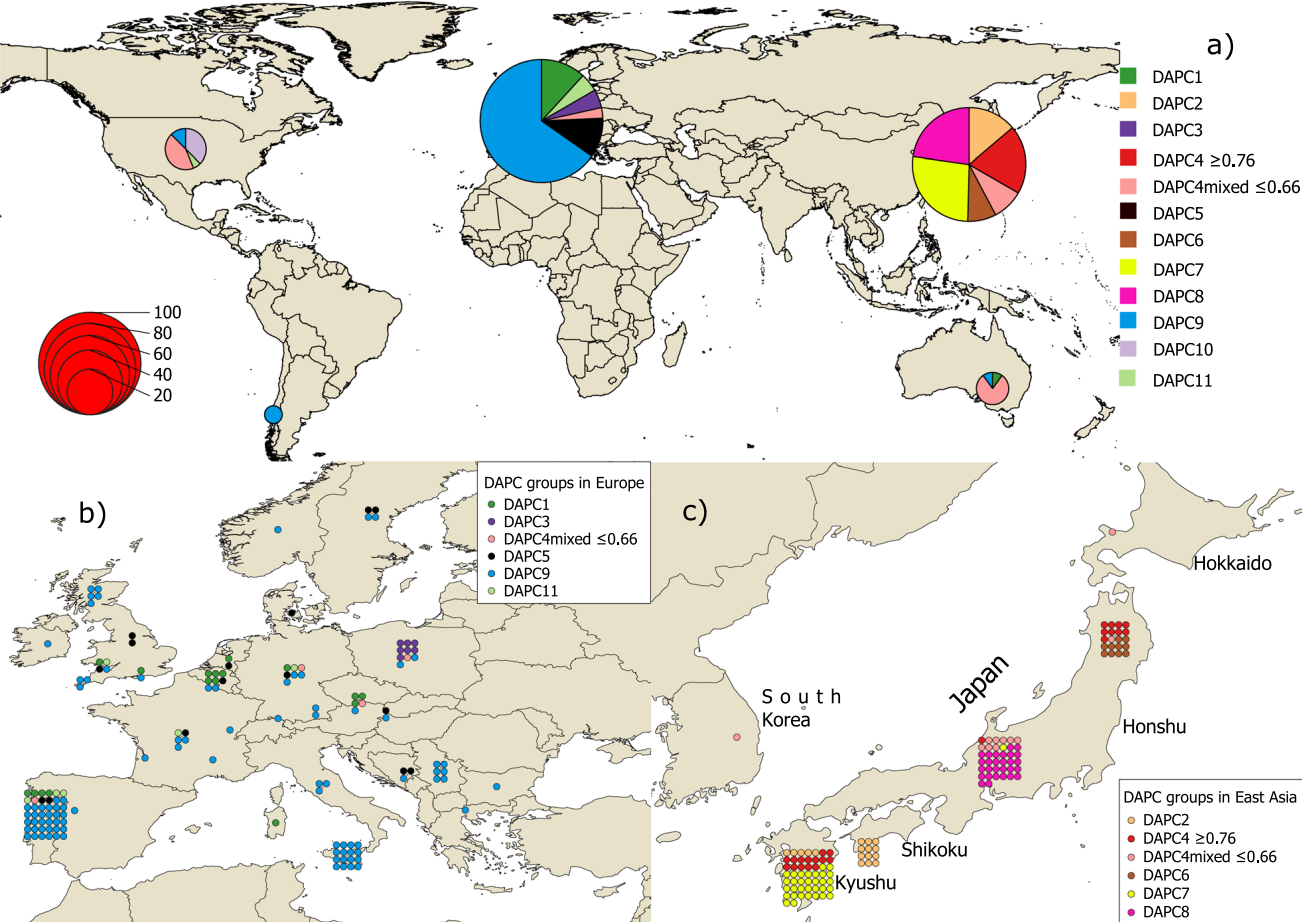
Distribution maps of *P.* × *cambivora* isolates and their DAPC groups. **a** Global overview with pie charts coloured by DAPC group. Size of pie chart corresponds to the number of isolates. **b** European sampling locations with each isolate represented by a dot coloured by its DAPC group. **c** East Asian sampling locations with each isolate represented by a dot coloured by its DAPC group. Multiple isolates from the same site are represented as a grid of dots centred on the sampling location

Global populations of *P.* × *cambivora* were highly structured by geographic region (Fig. 1). Most population groups were confined to a single continent, yet three population groups (DAPC1, 4mixed, and 9) were intercontinental and together made up the majority of isolates from Europe, North America and Australia (Fig. 2). The STRUCTURE analysis revealed clear, multilevel clustering with support for hybrid clusters, probably intraspecific hybrid clusters (Fig. 1). Preliminary assessment of delta K suggested only two clusters (Additional file 2: Fig. S1) which split a main group of *P.* × *cambivora* isolates from non-Asian regions (Europe, North and South America, and Australia), including the neo-type of the species, from a group of Asian and non-Asian isolates. However, based on the geography and prior knowledge of hybridization higher values of K were investigated (Additional file 3: Fig. S2, Fig. 1). The most informative number of clusters was five with distinct clusters apparent; increasing the number of clusters beyond this led to artificial splitting of single individuals into two clusters. Some isolates were admixed at all values of K and a number of admixed isolates formed fixed groups (e.g. DAPC5, DAPC11) and had stable admixture ratios.

The PCA (Additional files 4 and 5: Figs. S3 and S4), K-means clustering and assessment of the BIC from the DAPC analysis (Additional file 6: Fig. S5), and ML tree (Additional file 7: Fig. S6), revealed clear groups of isolates corresponding to those of the STRUCTURE results (Fig. 1), yet the DAPC groups split one of the STRUCTURE clusters into subgroups (DAPC groups 2, 3, 4, 6, 10). As all clustering methods produced similar groupings, the DAPC group names, which provided the highest level of substructuring, were retained for ease of reference. The sole exception to this was DAPC4 which in the STRUCTURE analysis showed consisted of some ‘pure’ isolates with a high membership probability to the group and some highly admixed isolates with a much lower membership probability to the group, with a clear gap in membership probabilities between these subgroups (i.e. no isolates with a membership probability > 0.66 and < 0.76). Therefore, the DAPC4 group was split into the more ‘pure’ DAPC4 (i.e. membership probability to STRUCTURE cluster 1 ≥ 0.76) and DAPC4mixed (i.e. membership probability to structure cluster 1 ≤ 0.66) solely for ease of visualization of the groups and results (Fig. 1).

### Sexual populations are found in Asia, whereas North American, Australian, and European populations are predominantly clonal

All isolates were self-sterile and produced oogonia with one of the two tester strains (A1 mating type isolate TJ0030 from DAPC1 and A2 isolate TJ0029 from DAPC9). Many groups consisted of a single mating type (Additional file 1: Table S1). DAPC1, DAPC7, DAPC8, and DAPC11 consisted entirely of A1 isolates (except for a single isolate in DAPC11 forming oogonia in pairings with both mating types). In contrast, DAPC5 and DAPC9 consisted entirely of A2 isolates. Groups DAPC2, DAPC3, DAPC4 (both subgroups), DAPC6, and DAPC10 contained both A1 and A2 isolates; these groups are closely related (see Fig. 1, Additional file 7: Fig. S6) and have a significant contribution from STRUCTURE cluster 1 (orange in Fig. 1).

**Fig. 3:**
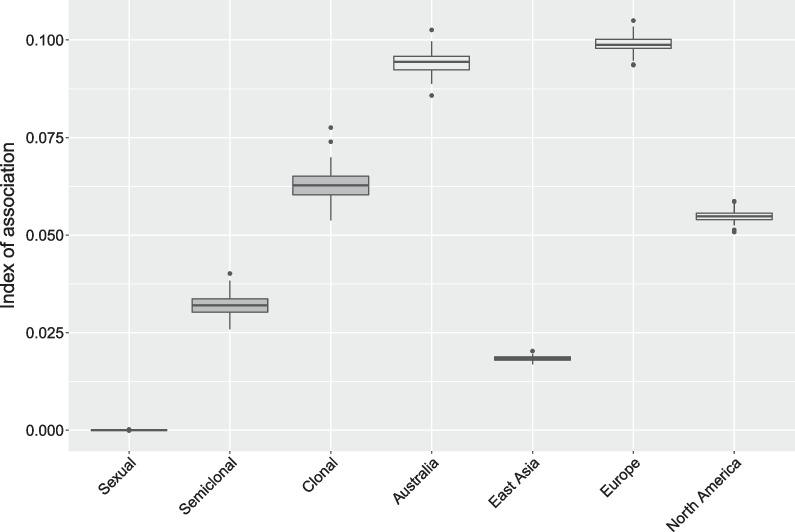
Estimation of the degree of linkage disequilibrium by the index of association (I_A_) in *P.* × *cambivora* populations. The first three boxplots represent the I_A_ for simulated populations under sexual, semiclonal, and clonal reproduction. All groupings were significantly different based on the Kruskal–Wallis rank sum test

The regional tests for linkage disequilibrium showed that the European and Australian populations reproduced clonally (Fig. 3). The North American population was highly clonal, yet indicated limited sexual reproduction occurs, as the I_A_ was lower than that of the simulated data for a purely clonal population and strongly deviated from the European and Australian populations. In contrast the I_A_ of the East Asian population was between a semiclonal and purely sexual population, i.e. it reproduced partially sexually.

### Recent and ancestral sexual hybridization are evident

**Fig. 4:**
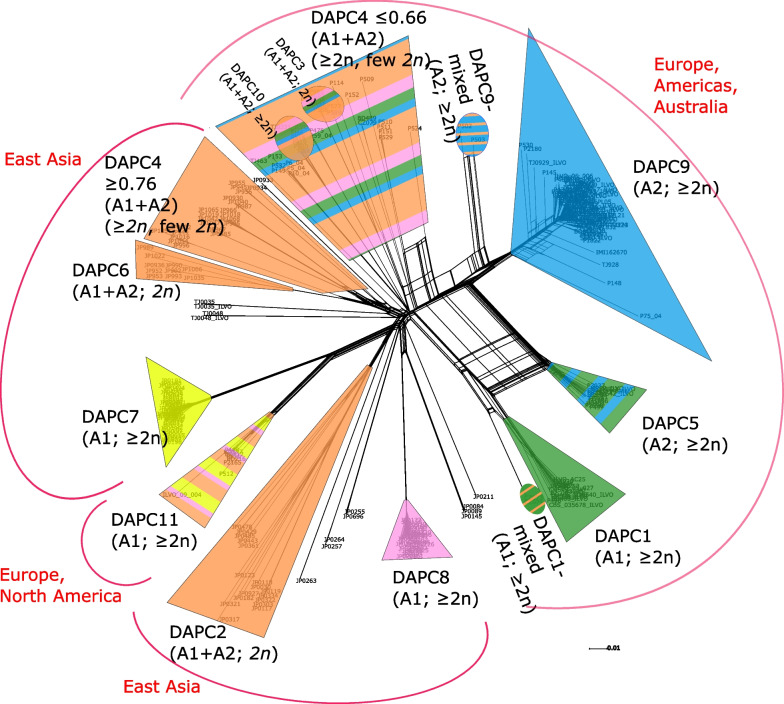
Splitstree network of the *P.* × *cambivora*-related isolates constructed with the equal angle algorithm using uncorrected p-distances. DAPC groups are outlined and coloured by their STRUCTURE membership probabilities. In parentheses below the DAPC group label are the mating type and ploidy level of the group

The Splitstree network analysis (Fig. 4) revealed similar patterns to those of the population clustering analyses while highlighting gene exchange and the intraspecific hybrid nature of some groups (e.g. DAPC5) and isolates (represented by boxes in the network).

**Fig. 5:**
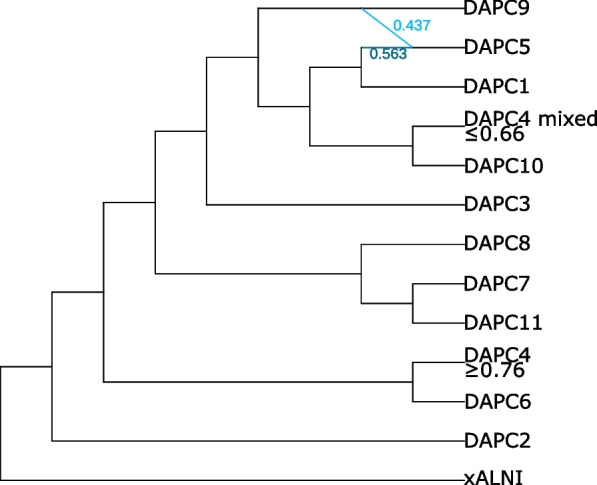
Phylogenetic network of *P.* × *cambivora*-related population groups estimated with the Species Networks applying Quartets (SNaQ) implemented in Phylonetworks with one hybridization event. The blue edges denote the identified hybridization event, with numbers next to the edges denoting the proportion of loci that were transferred from each lineage

The SNaQ results indicated a bifuricating tree was a poor fit to the data. The pseudolikelihood increased sharply from h = 0 to h = 1, while increasing the number of hybridization events above two resulted in small (from 2 to 3 hybridization events) or negligible (> 3 hybridization events) increases in pseudolikelihood values (Additional file 8: Fig. S7). This suggests that the best-fitting phylogenetic model involved one hybridization event. The hybrid group is DAPC5 with contributions from DAPC1 and DAPC9 (Fig. 5). The contribution of DAPC9 to the hybrid DAPC5, γ = 0.437, in the Phylonetworks result is similar to the contribution of DAPC9 to DAPC5 in the STRUCTURE results (i.e. mean membership coefficient of 0.385).

The HyDe results are striking in the number of significant hybrid groups found (Additional file 9: Table S2). The clearest, most supported hybrid (the highest Z-score of 39.885) is DAPC5 with parental groups DAPC1 and 9 with a γ of 0.4. This γ value is similar to the STRUCTURE ancestral membership probabilities and the Phylonetworks γ value. Of note is that DAPC9 (*P.* × *cambivora* neo-type group) was also very often classed as a hybrid population (Z-score 15.119–4.879). Noteworthy is that groups DAPC2, 6 and 3 were never classed as hybrid groups.

### Evidence of variable ploidy levels

**Fig. 6:**
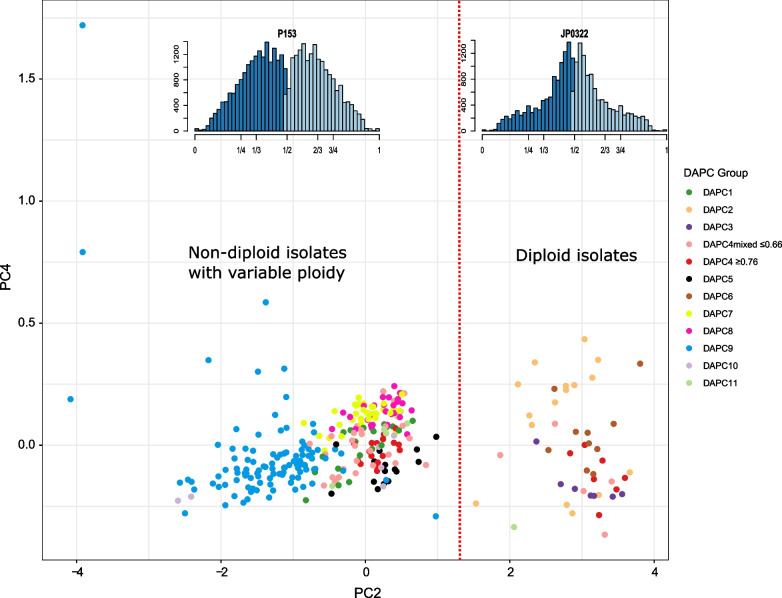
Principal components analysis of allelic ratios of heterozygous SNPs using gbs2ploidy showing two ploidy levels are present in the set of *P.* × *cambivora*-related isolates. The vertical dashed line separates diploid isolates (on the right) from non-diploid isolates (on the left). Inset plots show the distribution (histogram) of allele balance values for two example *P.* × *cambivora* isolates. JP0322 (DAPC2) shows a typical diploid plot; P153 (DAPC4mixed ≤ 0.66) shows a typical triploid plot. The frequency of the most abundant heterozygous allele is displayed in light blue, the frequency of the second most abundant heterozygous allele is displayed in dark blue. Expectations of the allele balance are displayed on the x-axis

Although the inference of ploidy analysis based on inferred ratios of minor and major allele frequency using read depth data for each isolate was not well resolved, there is evidence of variable ploidy. Two ploidy levels were apparent from the gbs2ploidy analysis (Fig. 6). Groups DAPC2, 3, and 6, together with a few isolates of DAPC4 (both subgroups) formed one ploidy group, with all other isolates falling into the second ploidy group. The plots of allele ratios indicated that the isolates in DAPC groups 2, 3 and 6 were diploid, having a clear peak at a 1:2 allelic ratio (inset Fig. 6). All other groups were potentially polyploid or aneuploid. Most isolates had a broad peak with an unclear ploidy level (Additional file 10: Fig. S8), although some isolates had peaks close to a 1:3 ratio suggesting triploidy (inset Fig. 6).

## Discussion

Our work provides novel insights into the global phylogeography and evolutionary history of *P.* × *cambivora*. Populations were highly structured by continent. The greatest diversity of groups was found in Japan, where both mating types also occurred. A comparison of simulated and observed index of association values suggests that reproduction in Japan is partially sexual, albeit with an important clonal component. Such a pattern would be expected in a native oomycete population that reproduces both sexually via oospores and asexually via zoospores. Together with the higher diversity of groups, it indicates that Japan lies within the centre of origin of *P.* × *cambivora*. Furthermore, the higher resistance of Asian chestnut species (*Castanea crenata* and *C. mollissima)* and hybrids between Asian and European chestnut to ink disease (Cristinzio and Grassi 1993; Salesses et al. 1993; Pereira et al. 1995; Fernández-López et al. 2001), consistent with co-evolution of Asian chestnuts with *P.* × *cambivora,* also indicates temperate Asia is the origin of the pathogen.

In contrast, populations in Europe, Australia, and North America were dominated by three clonal lineages and reproduced clonally, with apparently no, or only very limited, sexual reproduction. A highly diverse, sexually reproducing population is often a characteristic of pathogen populations at their centre of origin. When introduced elsewhere they often undergo genetic bottlenecks, resulting in a small number of clonally reproducing lineages, particularly *Phytophthora* pathogens (cf. Brasier 1995; Goodwin 1997), though these patterns may become altered by additional introductions and by recombination events. The devastating late potato blight pathogen, *P. infestans*, exemplifies this pattern with a diverse, sexual population in Mexico, its probable centre of origin, while elsewhere clonally reproducing lineages cause considerable economic damage (Goss et al. 2014; Hansen et al. 2016; Knaus et al. 2020). Similar patterns occur in the forest dieback pathogens *P. cinnamomi* and *P. ramorum*, where natural populations at their centre of origin in East and Southeast Asia are highly diverse and partially sexual, containing both mating types, and the panglobal invasive lineages are clonal (Goss et al. 2009; Van Poucke et al. 2012; Shakya et al. 2021; Jung et al. 2021).

The North American and Australian populations of *P.* × *cambivora* were principally composed of a diverse, admixed group (DAPC4mixed) also found in Europe and East Asia. The occurrence of the group across so many continents attests to its success as an invasive pathogen. However, isolates of this group were not found on chestnut species, but were common on fruit trees (*Prunus* and *Malus* spp.). Indeed most of the reports of *P.* × *cambivora* on fruit trees originate from the USA and Australia, as well as East Asia, not from Europe (Mircetich and Matheron 1976; Suzui and Hoshino 1979; Bumbieris and Wicks 1980; Wilcox and Mircetich 1985; Oudemans and Coffey 1991; Browne et al. 1995; Jee et al. 1997; Wicks et al. 1997). It therefore appears plausible that the dieback of fruit trees historically attributed to *P.* × *cambivora* is not due to the *P.* × *cambivora* lineages (i.e. DAPC9, DAPC1 and DAPC5) traditionally associated with ink disease in Europe, but to the related group DAPC4mixed (and possibly the closely related group DAPC4). Indeed, these latter groups could even constitute a separate sister species of *P.* × *cambivora*. A dataset from a larger group of *P.* × *cambivora* isolates from diseased fruit trees would be needed to thoroughly explore whether a separate taxon is responsible for the damage, together with host range and virulence assessments in comparative inoculation trials. Additionally, simpler identification of these groups would be highly desirable, e.g. using single or multi-locus barcodes based on Sanger sequences and/or morphological attributes.

The majority of isolates causing ink disease on chestnut and dieback of fagaceous tree hosts belonged to two clonal lineages (DAPC1 and DAPC9), each of an opposite mating type, and a clearly distinguishable hybrid group (DAPC5) between these two lineages. The two parental groups (DAPC1 and DAPC9) are widespread, occurring in Europe and Australia (both groups) and in North and South America (only DAPC9). They are known to sporulate well and have survived for many years; thus, they have proven themselves to be successful and evolutionarily fit entities. The formation of a manifest hybrid group between them, with no backcrosses, together with their distinctiveness suggests the two parental groups are independent lineages separated by substantial evolutionary time, although evidently not long enough for barriers to sexual reproduction to arise. A similar situation has been described for *P. ramorum,* with each of the twelve known lineages comprising a single mating type and separated by up to *c*. 1.6 million years (Goss et al. 2009; Jung et al. 2021; Van Poucke et al. 2012). A number of these lineages have been independently introduced to Europe and North America where they are responsible for two of the most devastating recent forest epidemics, sudden larch death and sudden oak death, respectively (Brasier and Webber 2010; Grünwald et al. 2012; Van Poucke et al. 2012).

Although *P.* × *cambivora* reproduces mainly clonally in Europe, the occurrence of a hybrid group between the two principal clonal lineages on the continent indicates that some sexual outcrossing has occasionally occurred. Furthermore, the restriction of the hybrid group (DAPC5) to regions where both parental groups co-occur, and the fact that all members of the hybrid group appear to be first generational hybrids, indicates the hybridization event took place in situ. This situation suggests that the DAPC5 hybrid group is either relatively new, unstable, or has slightly reduced fitness when compared to the parental groups. It is unlikely for novel hybrids to have the same fitness as their parents, very often they have reduced fitness and perish, whilst occasionally they have increased fitness and persist. With plant pathogens in general the host is usually the site or niche where fitness differences will be critical (Brasier 2001). With *Ophiostoma novo-ulmi* in North America dominant clonal lineages recombine but the recombinants are apparently unable to compete in fitness with the clones (Milgroom and Brasier 1997; Brasier and Kirk 2000). A prominent example of increased fitness in a hybrid is *P.* × *alni,* which is much more aggressive to *Alnus* and, hence, more widespread and abundant than its parent species *P.* × *multiformis* and *P. uniformis* (Brasier and Kirk 2001; Husson et al. 2015; Jung et al. 2018b). Increased fitness is a pre-requisite for persistence of novel hybrids, otherwise they will be outcompeted by their parents unless separated by geography, ecological niche, or a genetic barrier. An assessment of the relative fitness and virulence of DAPC1, DAPC9, and DAPC5 on their main tree hosts would allow a more detailed appraisal of their threat to forests.

Given that there is prior evidence that *P.* × *cambivora* is a hybrid (Jung et al. 2017b; Van Poucke et al. 2021) it was expected that many isolates in this study would also be of hybrid origin. However, the only undisputed hybrid group with both parents known was DAPC5; although many groups displayed evidence of admixture they were not confirmed as hybrids with known parents. Detection of hybridization using phylogenetic invariants revealed significant results for many of the triplets (non-negligible γ values from 0.3 to 0.6), and such a large number of significant triplets often indicates ancestral hybridization, with the signal of admixture retained in many of the groups (Blischak 2021). Ancestral hybridization events negatively affect γ estimates and spurious results are known to occur if hybrids are included as parents (Blischak and Kubatko 2019; Kong and Kubatko 2020). Therefore, although it is evident that hybridization, most likely in the form of sexual outcrossing, has played a crucial role in the evolutionary history of *P.* × *cambivora*, the parental taxa were not in this study. They may exist in unsampled areas elsewhere in East Asia. Although extensive surveys in Taiwan and Vietnam found no *P.* × *cambivora*-related isolates in natural ecosystems (Jung et al. 2017a, 2020) large areas of temperate China remain to be explored for *Phytophthora* diversity and could harbour additional *P.* × *cambivora*-related groups.

The high ploidy level of many of the groups is also consistent with an ancestral hybrid origin of *P.* × *cambivora*, as polyploidy is linked to ancient hybridization events (Bertier et al. 2013). Both Jung et al. (2017b) and Van Poucke et al. (2021) found evidence of polyploidy in *P.* × *cambivora* yet were unable to confirm the ploidy level of the species. Although some groups are clearly diploid (DAPC2, DAPC3, DAPC6) and never occur as hybrid groups in the hybridization analysis, variable ploidy is suggested in most of the other groups. Polyploids often exhibit a shift in ecological tolerances and seem to be more frequent in human-disturbed, competitive habitats than their diploid relatives whilst also having a greater potential for habitat colonization and expansion into novel niches (Baduel et al. 2018; Ehrendorfer 1980; Otto and Whitton 2000; Pandit et al. 2006; Soltis and Soltis 2000). Thus polyploidy can infer a fitness advantage and increased adaptability, and, in some environments, has been shown to accelerate evolutionary adaptation (Ramsey 2011; Selmecki et al. 2015; Baduel et al. 2018). Aquatic habitats provide conditions for continuous asexual reproduction and spread of oomycetes via zoospores and thus decrease the need for long-term survival and genetic adaptations to host populations and changing environmental conditions via sexually derived oospores (Brasier et al. 2003; Jung et al. 2011). Apparently, aquatic conditions also facilitate allopolyploid hybridizations and confer selective advantages for hybrids, as demonstrated by the abundance of allopolyploid hybrids from *Phytophthora* Clades 6, 7a and 9 in river systems of Chile, South Africa, Taiwan, Vietnam and Western Australia (Hüberli et al. 2013; Nagel et al. 2013; Oh et al. 2013; Burgess 2015; Jung et al. 2017a, b, 2018a, 2020). In the present study, many *P.* × *cambivora*-related isolates in Japan, Portugal and Chile also were recovered from forest streams.

Nonetheless, whole genome duplication and polyploidy can result in developmental disruption, not least in meiosis therefore many polyploids are restricted to vegetative or other forms of asexual reproduction (Otto and Whitton 2000; Schinkel et al. 2016; Herben et al. 2017; Baduel et al. 2018). This is particularly suitable for a pathogen undergoing rapid range expansion, with major disease epidemics often associated with prolific asexual reproduction (Ashu and Xu 2015; Drenth et al. 2019). This is also the case for *Phytophthora infestans*, with Knaus et al. (2020) revealing that typically, major late blight epidemics of potato are caused by triploid, clonally reproducing lineages, as opposed to diploid sexually reproducing populations at the pathogen’s centre of origin. Intraspecific variation in ploidy, as well as copy number variation, were also reported in other *Phytophthora* species (Bertier et al. 2013; Barchenger et al. 2017; Knaus et al. 2020). Bertier et al. (2013) believe this increase in *P. infestans* ploidy level was due to hybridization between divergent genotypes of the species. Such a pattern may also fit the *P.* × *cambivora* populations presented in this study, e.g. with groups DAPC1 and DAPC9 becoming globally invasive polyploid clonal lineages, and the increased ploidy level in many of the groups due to hybridization between genotypes. Yet polyploidy is not without its challenges and in time many polyploids undergo diploidization (Hollister 2015; Baduel et al. 2018). However, different classes of genes and sequences are retained preferentially, with others more likely to be returned to diploid status, a feature known as ‘biased fractionation’ (Wendel et al. 2018). This phenomenon is known to have occurred in some *Phytophthora* species (Martens and Van de Peer 2010) and may account for the unclear ploidy levels of many of the *P.* × *cambivora* isolates. Thus, parts of the genome may be diploid and other parts triploid or tetraploid. The fact that *P.* × *cambivora* has a functional heterothallic breeding system and produces ample viable oospores, whereas most true triploids are effectively sterile, suggests the genome is not a full triploid. This is in keeping with the ancient hybridization events detected in most of the groups. Alternatively, heterokaryosis, having multiple genetically distinct nuclei in a cell, could be the cause of the ambiguous ploidy levels of many of the isolates. Heterokaryosis has been found in a range of oomycete and, specifically, *Phytophthora* species (Long and Keen 1977; Catal et al. 2010; Bertier et al. 2013; Fletcher et al. 2019) and indeed for some *P.* × *cambivora* isolates using flow cytometry (Jung et al. 2017b).

## Conclusions

This study indicates that the highly diverse, sexually recombining population of *P.* × *cambivora* in Japan is most probably endemic and lies within the centre of origin of the pathogen. Populations in Europe, Australia, and North America are dominated by a number of introduced clonal lineages. The finding that the majority of isolates causing ink disease of *Castanea* comprise a few clonal lineages may simplify management of the disease, as radically different genotypes are unlikely to arise, even though the direct parents of these groups were not found. Conversely, another group causing damage to fruit trees found in East Asia, North America, Australia and Europe could constitute a separate sister species to *P.* × *cambivora.* Further research is called for to compare the virulence on key hosts of the major *P.* × *cambivora* groups found, while strengthening biosecurity to prevent further global movement of these diverse groups. To partially address this issue a soil infestation pathogenicity trial including *Fagus sylvatica* and representative isolates from all 11 DAPC groups is currently underway. This study draws attention to the complex ploidy levels of *P.* × *cambivora* and the formative role ancient hybridization events have played in the history of this species. These traits have served the species well, enabling it to become a globally successful pathogen, and highlight the continued biosecurity threat this pathogen poses, particularly through recombination and hybridization between long separated groups.

## Supplementary Information

**Additional file 1: Table S1**. Details of *P.* × *cambivora*-related isolates used in this study including population group, geographic location, mating type, isolate codes used in other culture collections, and STRUCTURE membership probabilities for each K = 5 cluster.

**Additional file 2: Figure S1**. Delta K plot of the STRUCTURE analysis, showing K = 2 as the best clustering of isolates.

**Additional file 3: Figure S2**. Bayesian clustering of *P.* × *cambivora*-related isolates inferred using the programme STRUCTURE at K = 2, K = 3, K = 4, and K = 5. Each isolate is represented by a vertical line partitioned into coloured sections that represent the isolate’s estimated membership fractions in each cluster. Black lines separate isolates from different DAPC groups (see main text for details).

**Additional file 4: Figure S3**. Principal components analysis of *P.* × *cambivora* isolates. Only the first two principal components are shown, which explain 26.1% and 12.5% of the variance, respectively. Ellipse colours represent DAPC groups; the mating type of each group is given in parentheses. The barplot inset shows the percentage of variance explained by each principal component.

**Additional file 5: Figure S4**. Principal components analysis of *P.* × *cambivora*-related isolates displayed using the second and fourth principal components which more easily differentiates groups DAPC2, DAPC4mixed ≤ 0.66, DAPC4 ≥ 0.76, and DAPC6. Ellipse colours represent DAPC groups.

**Additional file 6: Figure S5**. Scatterplot of the discriminant analysis of principal components (DAPC) of *P.* × *cambivora*-related isolates. Individual isolates are represented by dots that are coloured by their DAPC group. At the bottom right, the PCA eigenvalues are represented, with the number of principal components used in the optimized analysis in black. At the top right, the Discriminant Analysis (DA) eigenvalues are displayed.

**Additional file 7: Figure S6**. Maximum likelihood tree of *P.* × *cambivora*-related isolates inferred using RAxML and 1,000 bootstraps. The tree was rooted using *P.* × *alni* as an outgroup (not shown). Coloured vertical bars represent the DAPC group colour used in other figures.

**Additional file 8: Figure S7**. Pseudolikelihood profile with increasing number of hybridization events (hmax) allowed, obtained with the Species Networks applying Quartets (SNaQ) pipeline.

**Additional file 9: Table S2**. Table of results of the population-level hybridization detection analyses conducted in HyDe. Only significant results are shown, with their p-value, Z-score and Gamma value.

**Additional file 10: Figure S8**. Distribution (histogram) of allele balance values for all *Phytophthora* × *cambivora* isolates by DAPC groups. The frequency of the most abundant heterozygous allele is displayed in light blue, the frequency of the second most abundant heterozygous allele is displayed in dark blue. Expectations of the allele balance are displayed on the x-axis.

## Data Availability

Raw sequence reads are deposited in the NCBIs SRA (BioProject PRJNA896358). Custom scripts for pre-processing of the raw data were used from Van Poucke et al. (2021) and are available at https://gitlab.com/ahaegeman/GBS_Phytophthora and at Zenodo with https://doi.org/10.5281/zenodo.3363287.
