# Supplementary figures and images for: Phylogeography and population structure of the global, wide host-range hybrid pathogen Phytophthora × cambivora
Source: IMA Fungus. 2023 Feb 23;14:4. doi: 10.1186/s43008-023-00109-6 (PMC9951538; doi:10.1186/s43008-023-00109-6)

$$\text{DeltaK} = \text{mean}(|L''(K)|) / \text{sd}(L(K))$$

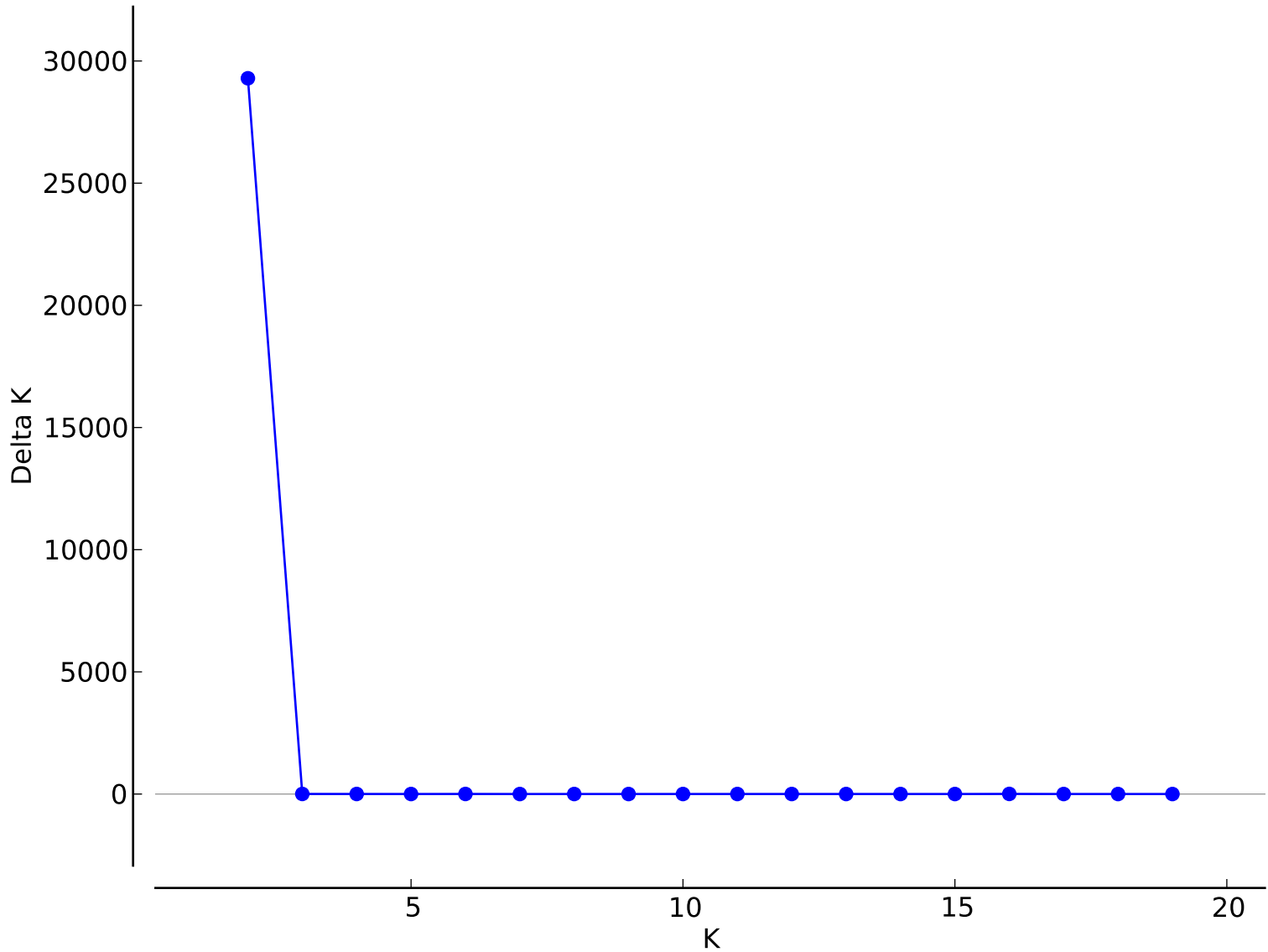

Supplement: Supplementary file 2 — Additional file 2: Figure S1. Delta K plot of the STRUCTURE analysis, showing K = 2 as the best clustering of isolates. [file 43008_2023_109_MOESM2_ESM.pdf]

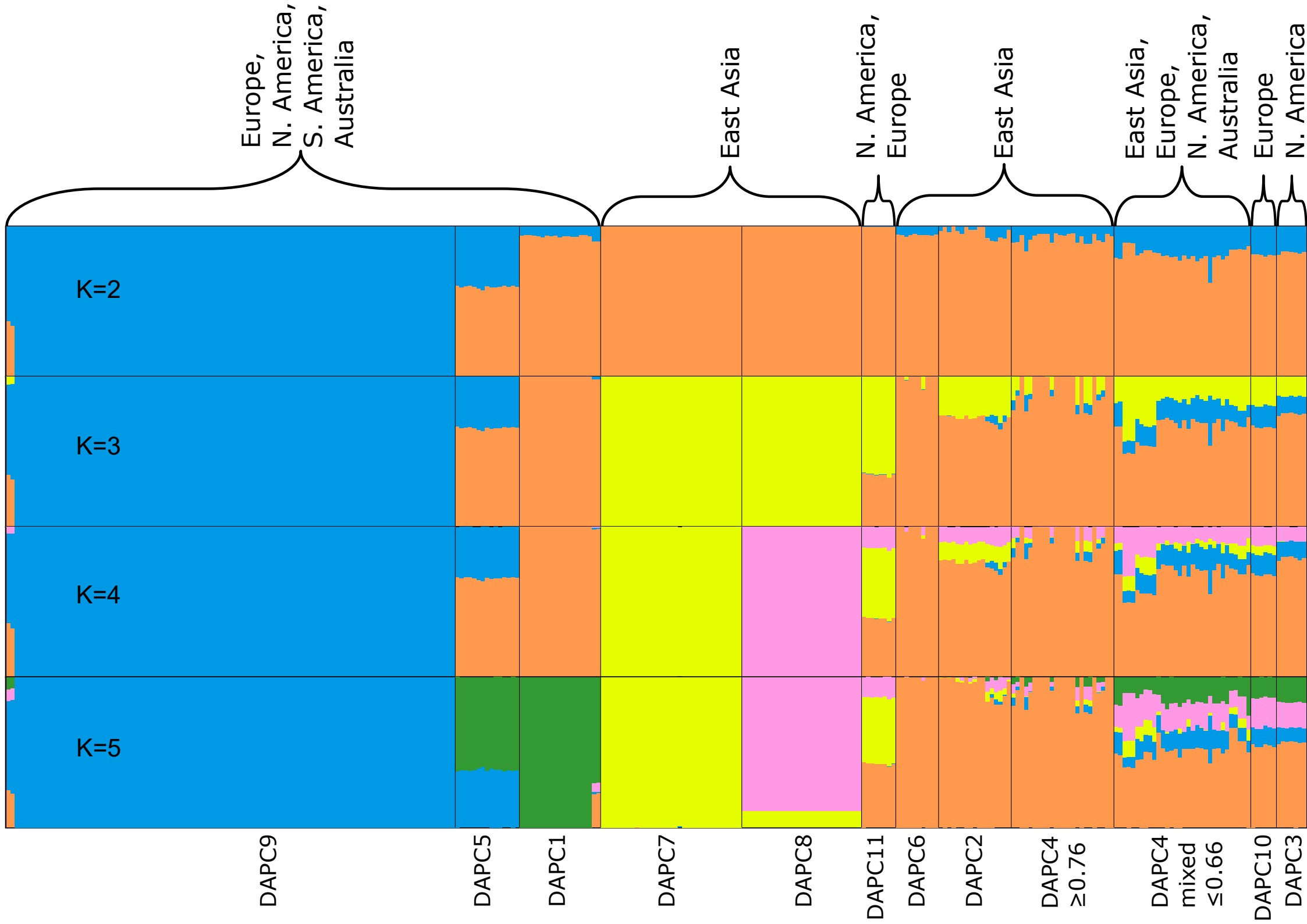

Supplement: Supplementary file 3 — Additional file 3: Figure S2. Bayesian clustering of P. × cambivora-related isolates inferred using the programme STRUCTURE at K = 2, K = 3, K = 4, and K = 5. Each isolate is represented by a vertical line partitioned into coloured sections that represent the isolate’s estimated membership fractions in each cluster. Black lines separate isolates from different DAPC groups (see main text for details). [file 43008_2023_109_MOESM3_ESM.pdf]

PC2

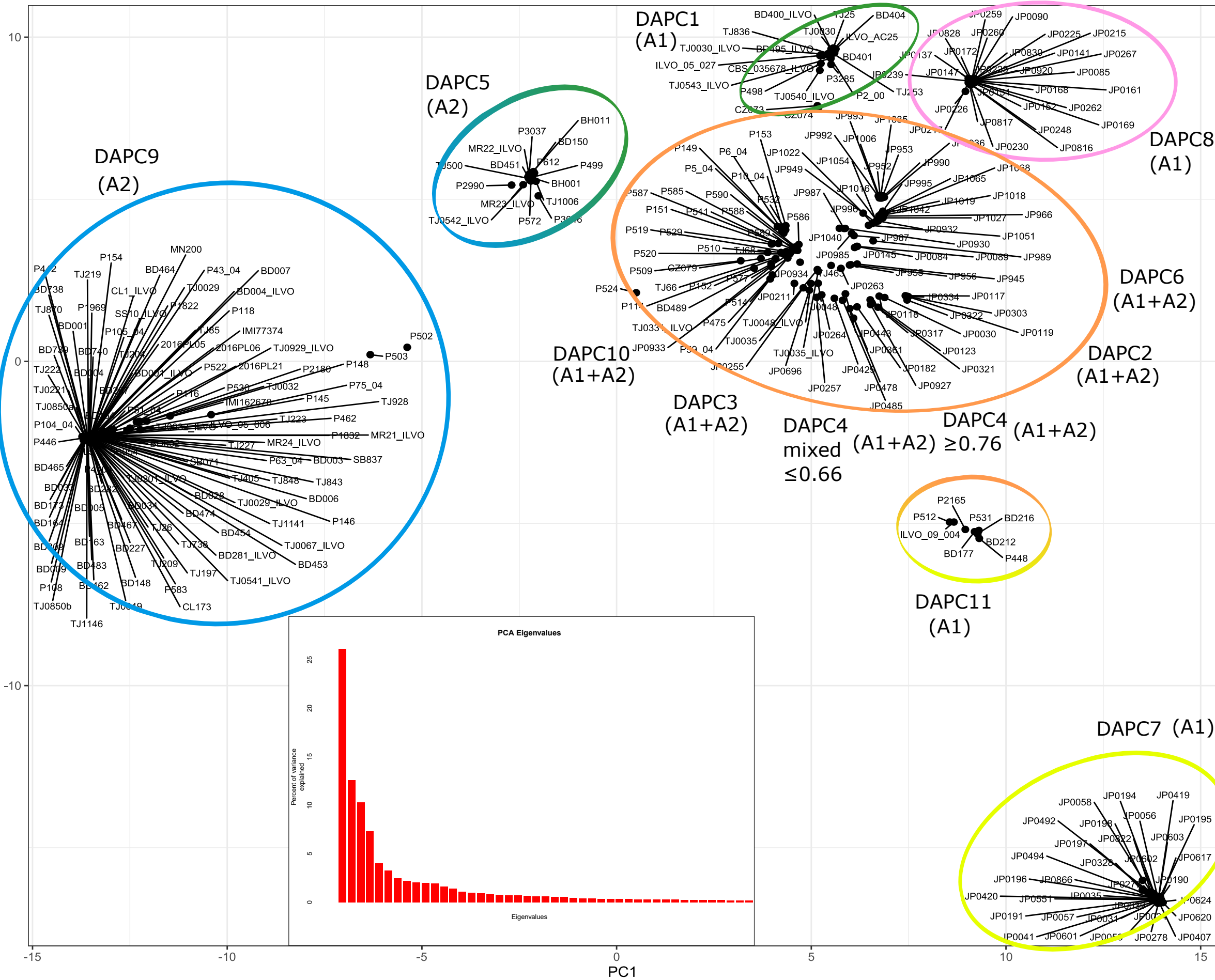

Supplement: Supplementary file 4 — Additional file 4: Figure S3. Principal components analysis of P. × cambivora isolates. Only the first two principal components are shown, which explain 26.1% and 12.5% of the variance, respectively. Ellipse colours represent DAPC groups; the mating type of each group is given in parentheses. The barplot inset shows the percentage of variance explained by each principal component. [file 43008_2023_109_MOESM4_ESM.pdf]

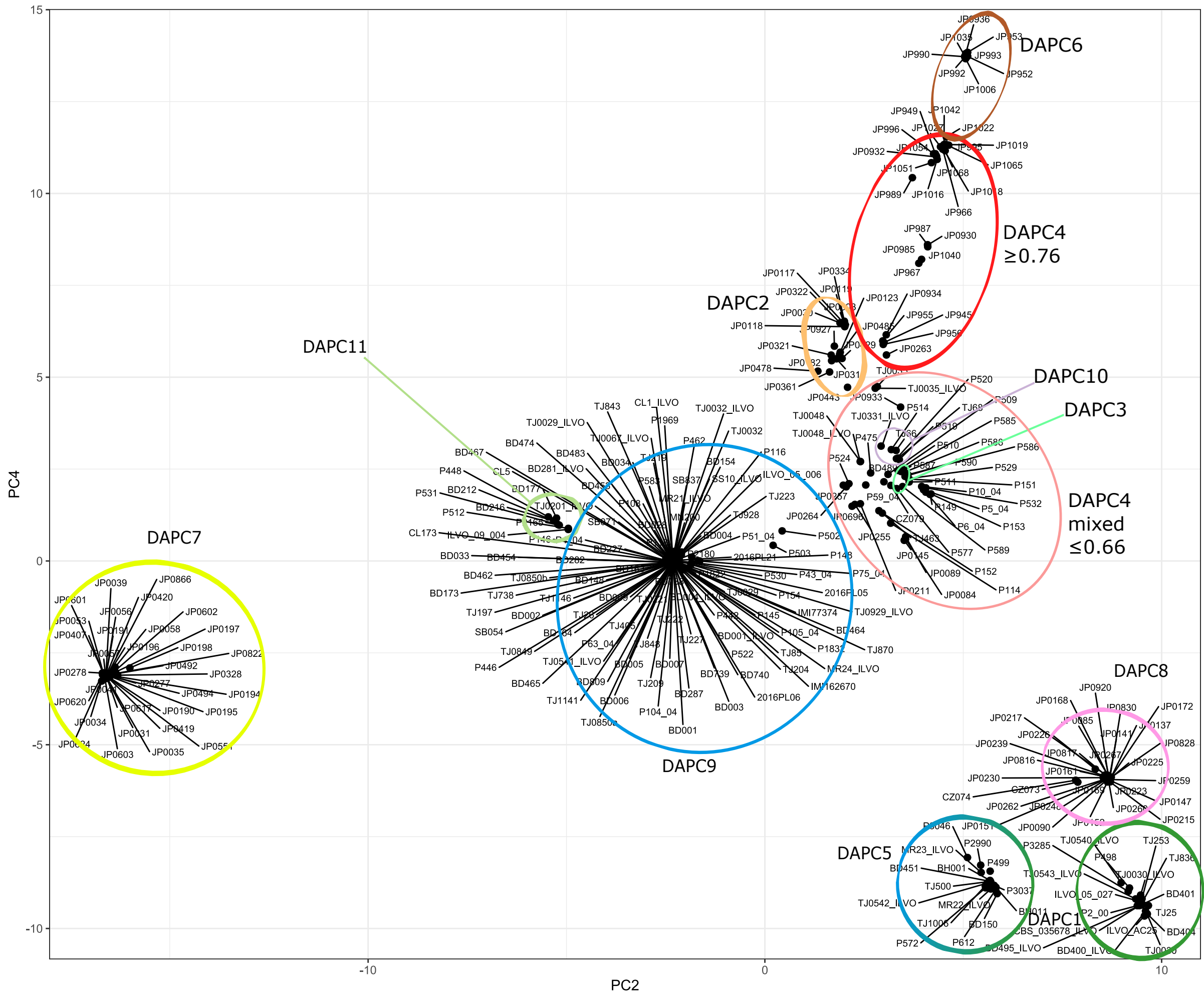

Supplement: Supplementary file 5 — Additional file 5: Figure S4. Principal components analysis of P. × cambivora-related isolates displayed using the second and fourth principal components which more easily differentiates groups DAPC2, DAPC4mixed ≤ 0.66, DAPC4 ≥ 0.76, and DAPC6. Ellipse colours represent DAPC groups. [file 43008_2023_109_MOESM5_ESM.pdf]

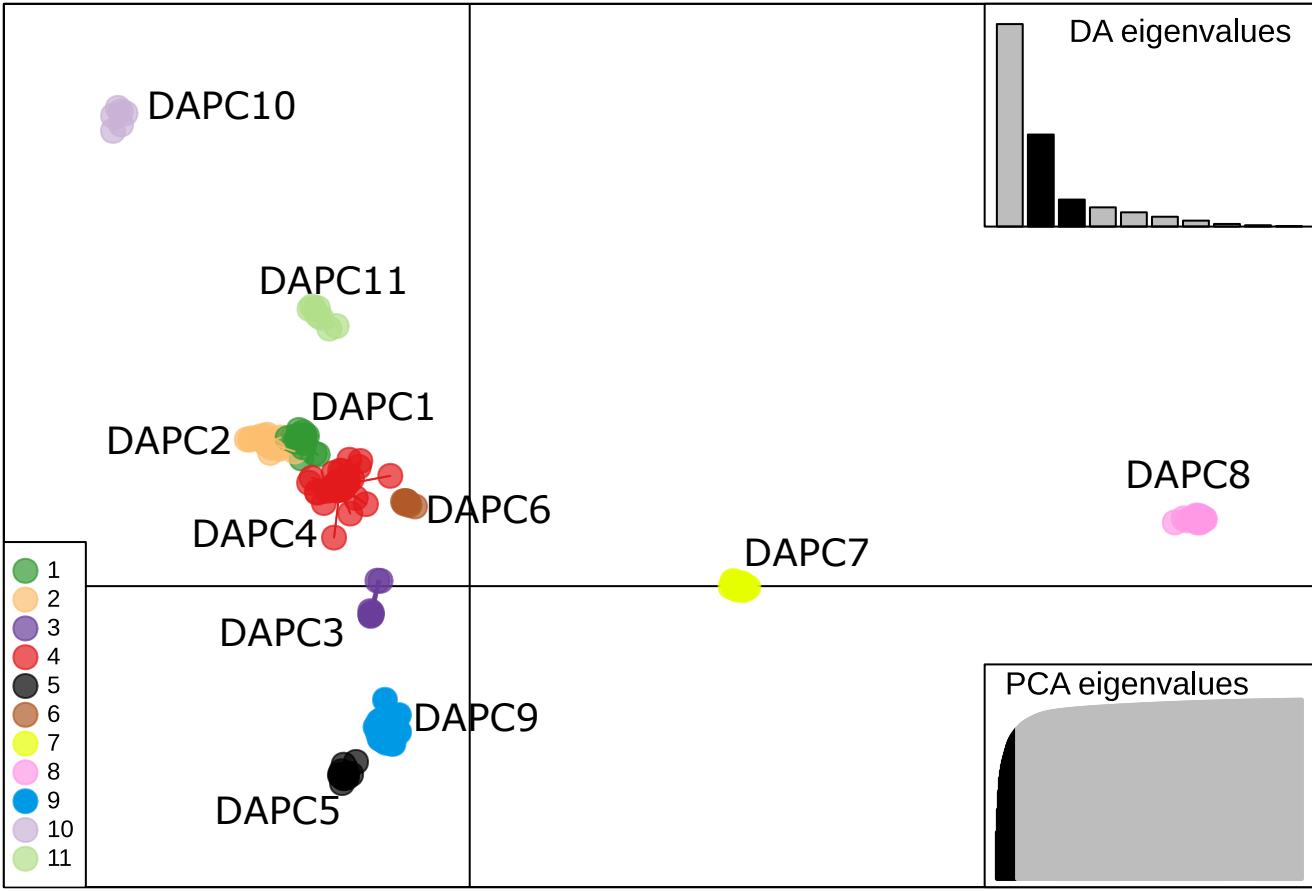

Supplement: Supplementary file 6 — Additional file 6: Figure S5. Scatterplot of the discriminant analysis of principal components (DAPC) of P. × cambivora-related isolates. Individual isolates are represented by dots that are coloured by their DAPC group. At the bottom right, the PCA eigenvalues are represented, with the number of principal components used in the optimized analysis in black. At the top right, the Discriminant Analysis (DA) eigenvalues are displayed. [file 43008_2023_109_MOESM6_ESM.pdf]

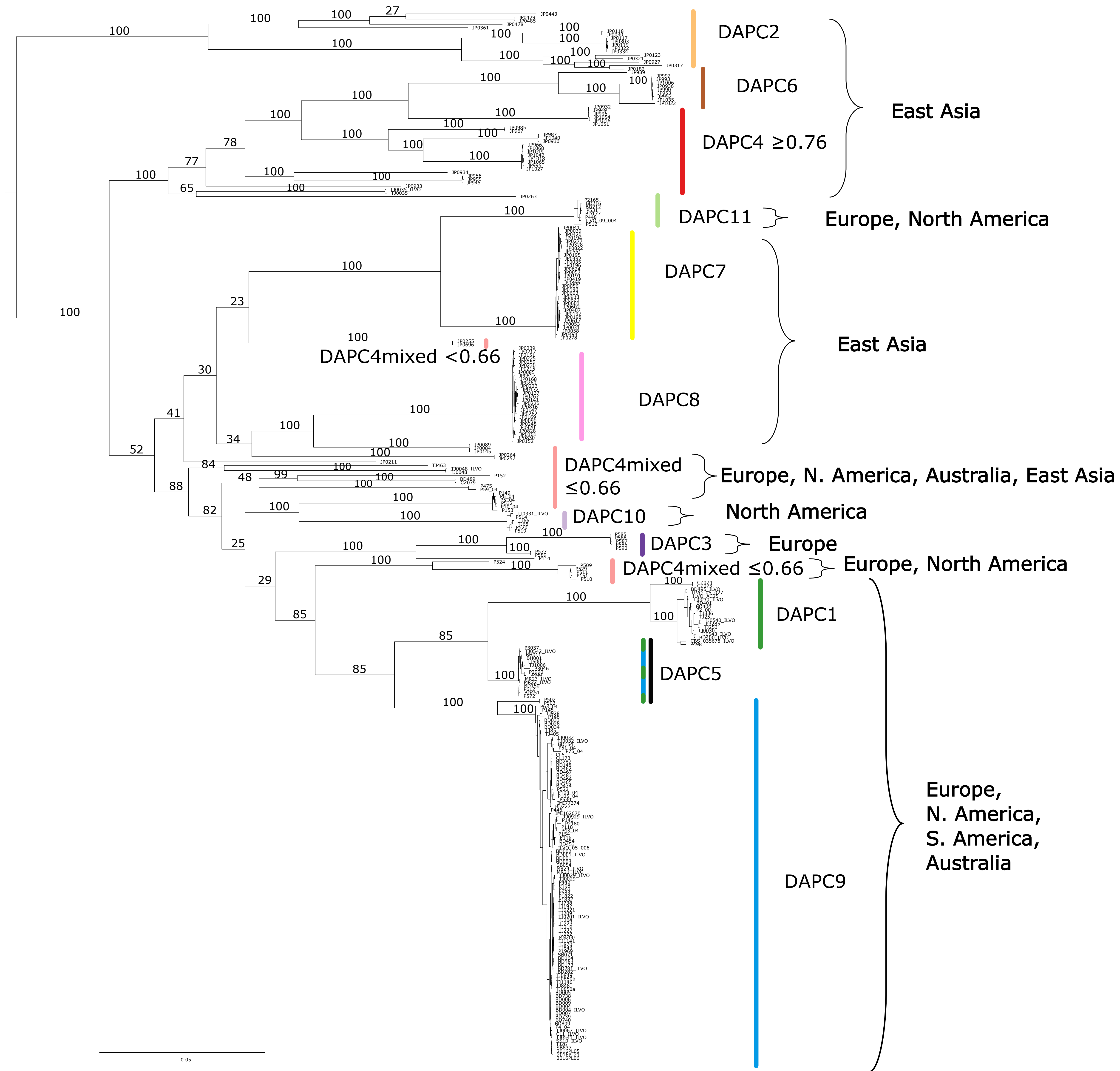

Supplement: Supplementary file 7 — Additional file 7: Figure S6. Maximum likelihood tree of P. × cambivora-related isolates inferred using RAxML and 1,000 bootstraps. The tree was rooted using P. × alni as an outgroup (not shown). Coloured vertical bars represent the DAPC group colour used in other figures. [file 43008_2023_109_MOESM7_ESM.pdf]

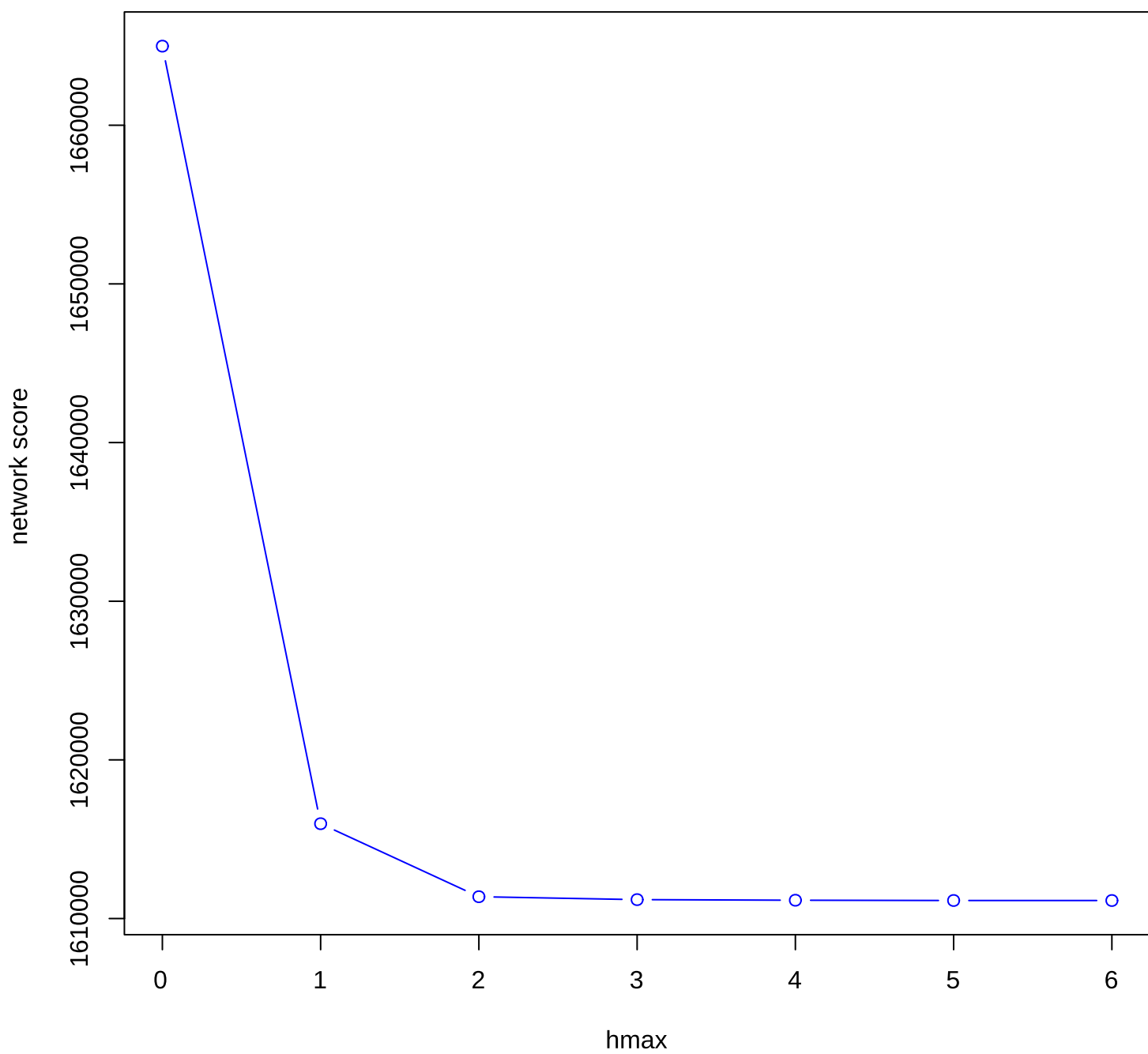

Supplement: Supplementary file 8 — Additional file 8: Figure S7. Pseudolikelihood profile with increasing number of hybridization events (hmax) allowed, obtained with the Species Networks applying Quartets (SNaQ) pipeline. [file 43008_2023_109_MOESM8_ESM.pdf]
