# Supplementary material for: Phylogeography and population structure of the global, wide host-range hybrid pathogen Phytophthora × cambivora
Source: IMA Fungus. 2023 Feb 23;14:4. doi: 10.1186/s43008-023-00109-6 (PMC9951538; doi:10.1186/s43008-023-00109-6)
Supplement: Supplementary file 10 — Additional file 10: Figure S8. Distribution (histogram) of allele balance values for all Phytophthora × cambivora isolates by DAPC groups. The frequency of the most abundant heterozygous allele is displayed in light blue, the frequency of the second most abundant heterozygous allele is displayed in dark blue. Expectations of the allele balance are displayed on the x-axis. [file 43008_2023_109_MOESM10_ESM.docx]

Additional File 10: Figure S8 Distribution (histogram) of allele balance values for all *Phytophthora ×cambivora* isolates

by DAPC groups. The frequency of the most abundant heterozygous allele is displayed in light blue, the frequency of

the second most abundant heterozygous allele is displayed in dark blue. Expectations of the allele balance are displayed

on the x-axis.

| DAPC1 | Page 2-3 |
| --- | --- |
| DAPC2 | Page 4-5 |
| DAPC3 | Page 6 |
| DAPC4 | Page 7-8 |
| DAPC4mixed | Page 9-11 |
| DAPC5 | Page 12 |
| DAPC6 | Page 13 |
| DAPC7 | Page 14-16 |
| DAPC8 | Page 17-18 |
| DAPC9 | Page 19-25 |
| DAPC10 | Page 26 |
| DAPC11 | Page 27 |

DAPC1

DAPC2

DAPC3

DAPC4

DAPC4mixed

DAPC5

DAPC6

DAPC7

DAPC8

DAPC9

DAPC10

DAPC11
